# Dynamic locking screws in proximal humeral plate osteosynthesis demonstrate superior fixation properties: a biomechanical study

**DOI:** 10.1186/s40634-020-00293-z

**Published:** 2020-10-12

**Authors:** Gunther Sandmann, Atesch Ateschrang, Thomas Freude, Ulrich Stöckle, Werner Schmölz, Christian Konrads, Stefan Döbele

**Affiliations:** 1 Sportsclinic Ravensburg, Ravensburg, Germany; 2 Department of Trauma and Reconstructive Surgery, BG Klinik, University of Tübingen, Schnarrenbergstr. 95, 72076 Tübingen, Germany; 3 Department of Orthopaedics and Traumatology, University of Salzburg, Salzburg, Austria; 4 Center for Musculoskeletal Surgery, Charité – University Medical Center Berlin, Berlin, Germany; 5 Department of Biomechanics, University of Innsbruck, Innsbruck, Austria

**Keywords:** Proximal humeral fracture, Humeral head fracture, PHILOS, Reosteosynthesis

## Abstract

**Purpose:**

Angular stable implants reduced the complication rate in the treatment of humeral head fractures. But the failure rate is still high. To further reduce the risk of cut-out, cement augmentation of screws was introduced. A reason for failure of plate osteosynthesis might be the extremely high stiffness of the screw-plate interface leading to a loss of reduction and cut-out of screws. A more homogeneous distribution of the forces on all screws may avoid secondary dislocation. We hypothesize that dynamic osteosynthesis minimizes screw loosening and results in a higher load to failure than standard locking screws.

**Methods:**

Twelve paired human humerus specimens were analysed. A standardized three-part fracture model with a metaphyseal defect was simulated. Within each pair of humeri, one was fixed with a Philos plate and standard locking screws (LS), whereas the other humerus was fixed with a Philos plate and dynamic locking screws (DLS). A cyclic varus-bending test or a rotation test with increasing loading force was performed until failure of the screw-bone-fixation.

**Results:**

In the varus bending test, pairs failed by screw loosening in the humeral head. The LS-group reached 2901 (601–5201) load cycles until failure, while the DLS-group failed after 3731 (2001–5601) cycles. This corresponds to a median loading of 195 N for the LS-group and 235 N for the DLS-group (*p* = 0.028). In the rotation test the LS-group reached a median of 1101 (501–1501) load cycles until failure of fixation occurred, while the DLS-group failed after 1401 (401–2201) cycles (*p* = 0.225).

**Conclusions:**

Plate fixation using dynamic locking screws for the treatment of proximal humerus fractures demonstrated more load cycles until failure compared to standard locking plate osteosynthesis.

## Background

Proximal humeral fractures can be treated using several surgical and non-surgical concepts [5, 7, 25]. For stable fractures a non-operative treatment is recommended [7, 8]. Three- and four-part fractures could be handled with operative stabilization techniques including minimally invasive osteosynthesis, open reduction and plate fixation, intramedullary nail osteosynthesis, or primary arthroplasty [4, 11, 14, 15]. To prevent failures, it is essential to choose the most suitable treatment [1, 22, 30].

**Fig. 1:**
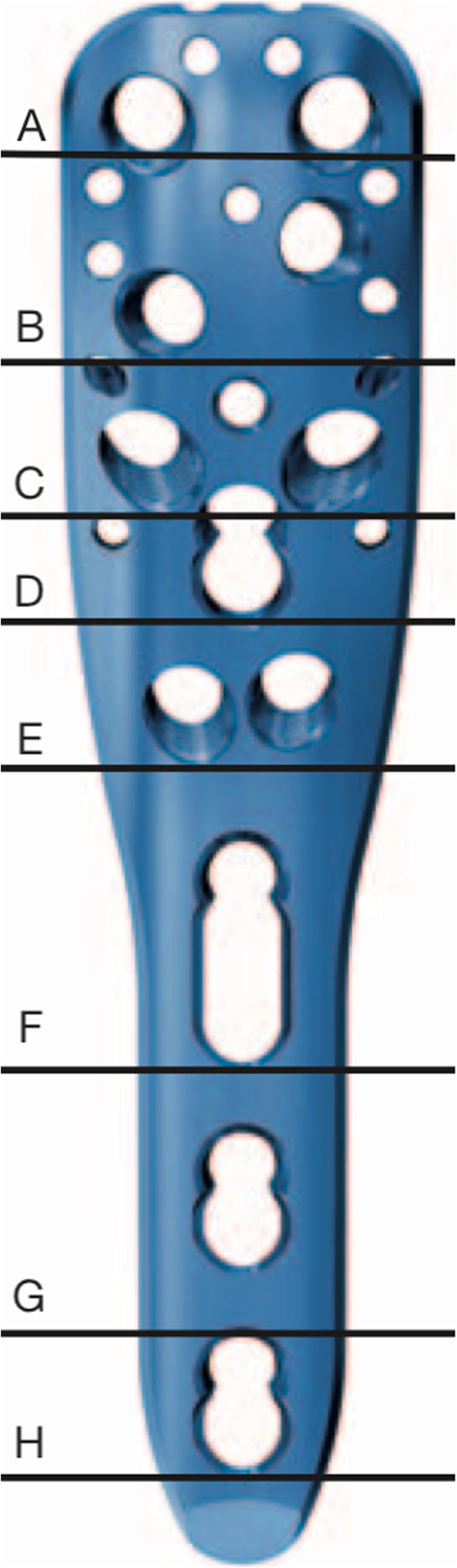
PHILOS plate with 3 shaft holes

The development and introduction of locking plates (Fig. 1) led to promising results [20]. However, the complication rate using locking plate osteosynthesis is still rather high in proximal humeral fractures. A multicenter study published in 2012 by Südkamp et al. encountered complications in 34% of 155 patients within 12 months postoperatively. The most common complication (14%) was screw perforation of the humeral head [27]. Other authors also reported a high screw perforation rate demonstrating that cut-out of screws was the most common complication within three postoperative months. A systematic review described an overall complication rate of 48.8%, including a revision rate of 13.8%. Varus malunion was observed in 16.3% of cases, osteonecrosis in 10.8%, intraarticular screw perforation in 7.5% [26].

Therefore, adaptions to the implants have been made and by the use of medial calcar support by a blade device, secondary varus displacement could be reduced [24]. In severely displaced fractures and reduced bone quality the use of allograft struts leads to improved results [6]. In a comparative clinical trial, augmentation with calcium phosphate cement significantly decreased (*p* = 0.02) displacement and intraarticular screw perforation in fractures with metaphyseal comminution [21, 24]. In a biomechanical study, Unger et al. observed the effect of in situ augmentation on implant anchorage in proximal humeral fractures [28]. Augmentation of cannulated screws might be a practicable method to enhance screw anchorage [23]. Indeed, the use of cement augmentation improves screw anchorage and thereby reduces loss of reduction [3, 17]. But there are still cases, where the cement augmentation is not favorable and here the dynamic osteosynthesis might be a better choice [13].

**Fig. 2:**
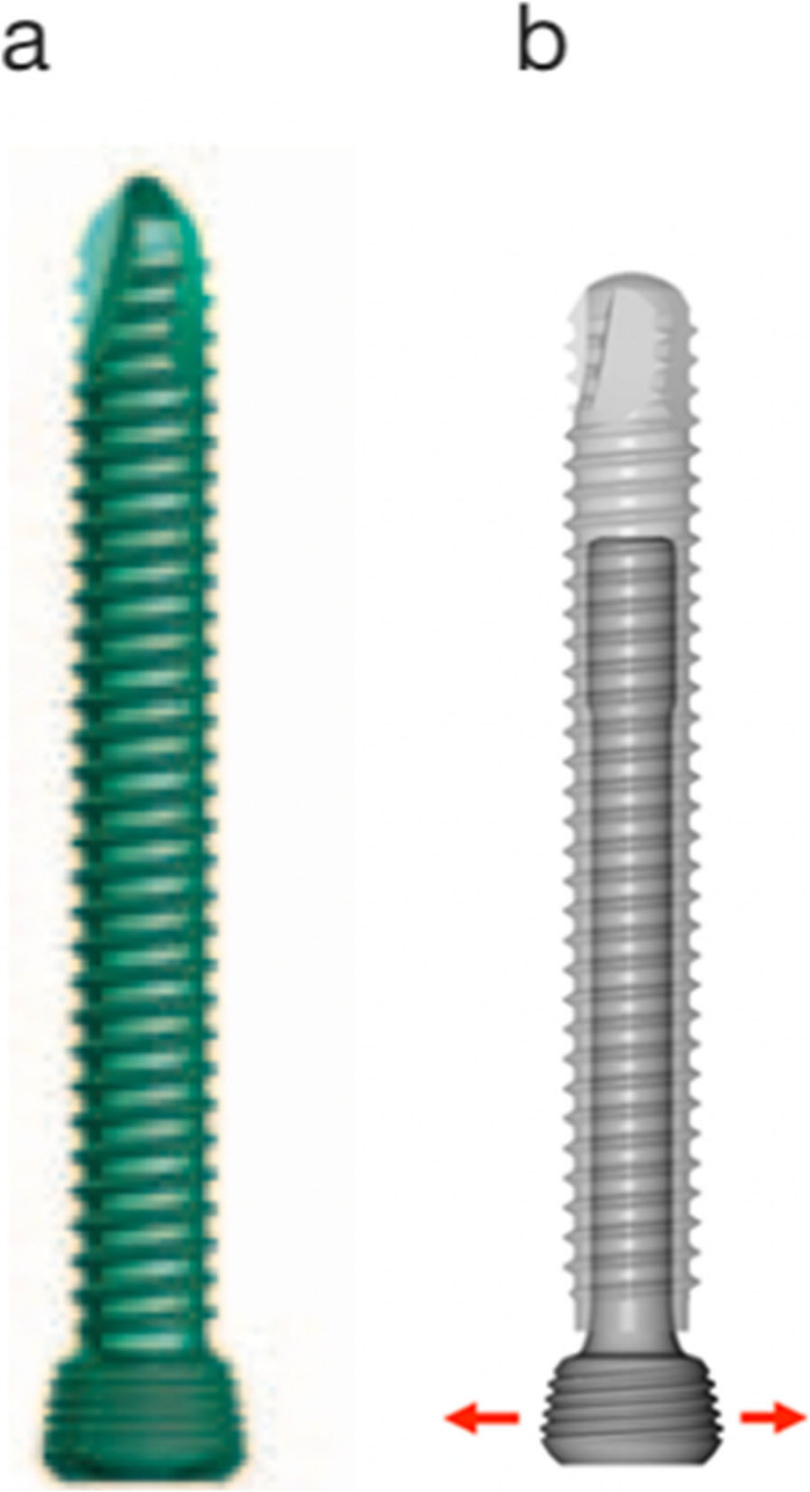
Screw types. **a** Standard locking screw, **b** Dynamic locking screw

Dynamic Locking Screws (DLS) are a further development of locking screws (Fig. 2). These screws allow for some movement of the outer part of the screw (threaded sleeve) relative to the core part (pin) of the dynamic head locking screw. These implants are used with standard locking plates. They are especially recommended in comminuted fractures treated with bridging plate osteosynthesis. Stiffness of the osteosynthesis is reduced, especially at the plate-sided parts of the fracture site, which usually is the tension side of the bone [9, 10]. Several studies demonstrated that DLS could act like a damper [12]. We suppose that this might be especially beneficial in weak bone like the humeral head. Generally, DLS lead to a reduction of force peaks and thereby better stress distribution over all screws. Using DLS, an increased screw anchorage in the bone might be achievable even without augmentation.

The aim of the present biomechanical study was to evaluate implant anchorage with DLS versus standard locking screws (LS) in plate osteosynthesis of three-part proximal humeral fractures under cyclic loading in varus bending and rotation tests.

## Materials and methods

A model of proximal humeral fracture plate osteosynthesis was used for ex-vivo biomechanical testing until failure by fragment dislocation. The applied test setup, loading protocol, and fracture model was adapted from a previously published study [28] investigating proximal humeral fracture plate osteosynthesis and is described in the following.

### Specimens

Twelve pairs (*n* = 24) of fresh frozen human cadaveric humeri (mean age 72.9 +/− 10.84 years, 8 females and 3 males) were used for the tests. The Bone-Mineral-Density (BMD) in the humeral head was assessed using the method described by Krappinger et al. [16]. Specimens were randomly distributed into four homogeneous groups regarding age, gender, and BMD. Within each pair of humeri one sample was randomised for DLS implantation.

A standardized three-part fracture of the humeral head with metaphyseal comminution was simulated. Fracture reduction and fixation was carried out with a PHILOS plate osteosynthesis (DePuy Synthes, Oberdorf, Switzerland, Fig. 1). For fixation of the PHILOS plate (Section A-C) 6 head locking screws (LS 3.5 mm, Fig. 2a) or 6 dynamic head locking screws (DLS 3.7 mm, Fig. 2b) were used. Plate and screws were implanted according to the manufacturer’s recommendations. In all test groups a short PHILOS plate with 3 shaft holes was used (Fig. 1). In all osteosynthesis the 3 shaft holes (Section F-H) were occupied with bicortical head locking screws (LS 3.5, Fig. 2a).

The screw length was determined by measuring the distance between the lateral and the medial cortex or the subchondral bone using the measurement tool provided by the manufacturer. In order to have comparable screw lengths the second humerus of a pair always received the same screw lengths, which were measured for the contralateral humerus of the pair.

All humeri were embedded in PMMA cement (Polymethylmethacrylat, Technovit 3040, Heraus Kulzer, Wertheim, Germany) to allow for fixation of the specimens in the material testing machine.

### Biomechanics of DLS

We measured the loading of screws and their load distribution in a locking plate osteosynthesis model of a long bone diaphyseal fracture using standard locking screws or dynamic locking screws. Then we used these results along with information about the distribution of bone mineral densitiy (BMD) of the humeral head to develope a finite elements model (FEM) for estimating screw loading and load distribution among the screws in a proximal humerus plate osteosynthesis using standard locking screws versus DLS.

### Testing

Varus bending and axial rotation tests were performed to evaluate biomechanical characteristics of both osteosynthesis (dynamic osteosynthesis vs. conventional osteosynthesis)**.** A biaxial servo-hydraulic material testing machine (MTS, 858 MiniBionix II, MN, USA) was used with a specifically designed loading jig and a cyclic loading protocol.

The different test groups were arranged in a pair wise fashion:

1. Bending test (*n* = 12)
  - standard locking screws (*n* = 6, PHILOS with LS)
  - dynamic locking screws (*n* = 6, PHILOS with DLS)
2. Rotation test (*n* = 12)
  - standard locking screws (*n* = 6, PHILOS with LS)
  - dynamic locking screws (*n* = 6, PHILOS with DLS)

At the proximal end of the PHILOS plate and at the minor tubercle, markers of the 3D motion analysis system (Winbiomechanics, Zebris, Isny, Germany) were fixed. The system recorded rotation and translation in all six degrees of freedom and by that the relative motion between the PHILOS plate and the humeral head were detected. For defining the coordinate system in the humeral head, landmarks were chosen to approximate the center of the coordinate system in the center of the humeral head.

**Fig. 3:**
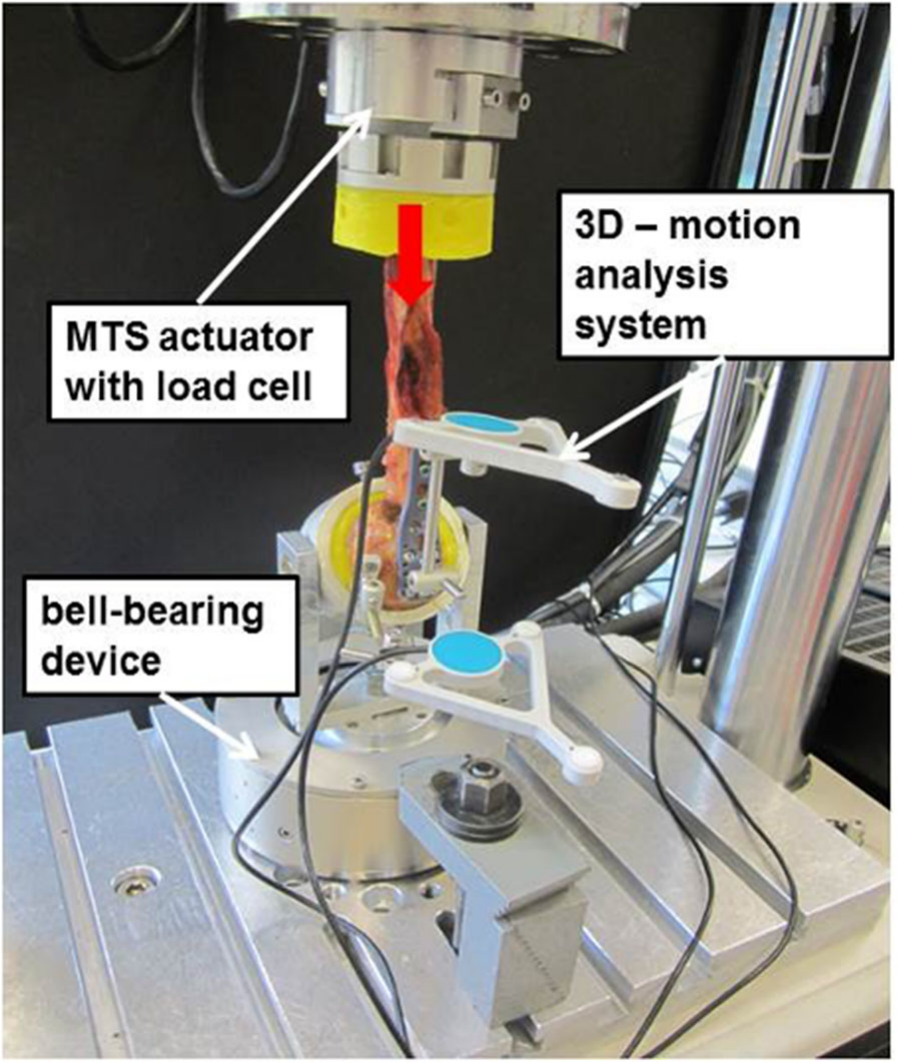
Setup for the varus bending test

For the bending test, the embedded distal humeral shaft was fixed to the actuator of the servo-hydraulic testing machine. The humeral head was fixed via a ball-bearing device to minimise shear forces (Fig. 3). Specimens were cyclically loaded until failure occurred. Initially, the loading ranged from + 10 N (tension) to -50 N (compression), while the load magnitude in compression increased stepwise by 5 N every 100 load cycles. The loading rates for all load magnitudes were 5 mm/sec. This resulted in a compressive load magnitude of 50 N after 100 load cycles, 100 N after 1000 load cycles, 150 N after 2000 load cycles etc. The bending tests were carried out in a cantilevered fashion to produce a varus (valgus-tension) bending moment. The lever arm was between the PHILOS plate and the point of load application and accounted for approximately 45 mm. This resulted in an approximate initial bending moment from 0.45 Nm in valgus to 2.25 Nm in varus. While the bending moment in valgus was kept constant, the bending moment in varus increased to 4,5 Nm after 1000 loading cycles and to 6,75 Nm after 2000 loading cycles. Specimens were cyclically loaded until obvious failure of the screw bone fixation. In the post testing data analysis, failure was defined as an increase of angular tilting in varus of more than 0.5 degrees within 100 load cycles.

**Fig. 4:**
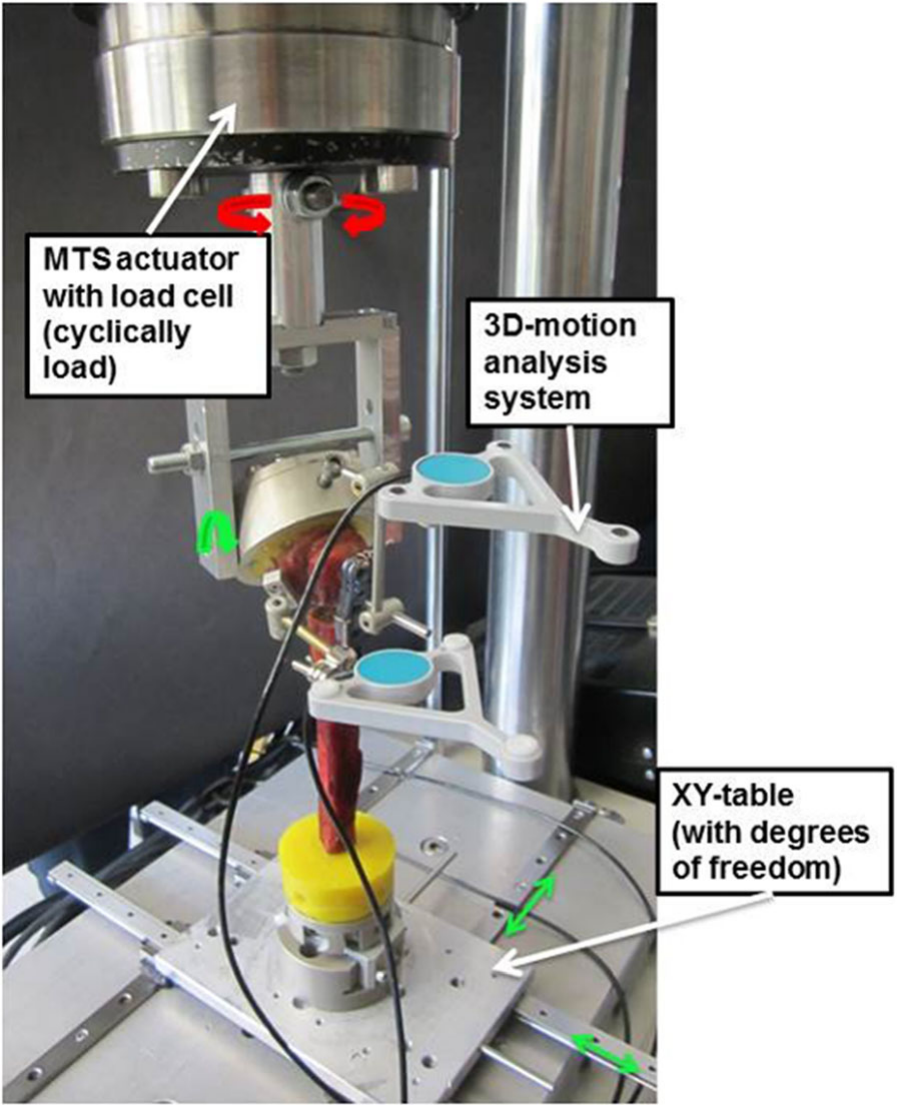
Test setup for loading in axial rotation. Red arrows indicate load application. Green arrows indicate free translations and free rotation

For axial rotation testing, the humeral head was fixed to the actuator of the servohydraulic testing machine while rotation in the anterior–posterior plane was allowed. The applied torque acted along the humeral shaft axis. The distal end of the humerus was fixed to a xy-bearing table to minimise shear forces (Fig. 4). Specimens were cyclically loaded with a constantly increasing torque and a constant axial load of 20 N. The sinusoidal torque was applied with 0.25 Hz and initially ranged from − 0.5 to + 0.5 Nm. With each load cycle, the range in both directions was increased by 0.0025 Nm, e.g. after 1000 cycles the torque ranged from − 3.0 to + 3.0 Nm. Every 100 load cycles, motion data was recorded and maximum rotation of the humeral head to the plate was determined. Specimens were loaded until failure of the screw/bone fixation. Failure was defined as an axial rotational motion larger than four degrees during one load cycle.

### Statistical analysis

The numbers of failure cycles by the standard LS 3.5 mm were compared with the numbers of failure cycles by the DLS 3.7 mm screws (in varus bending test and in axial torsion test). Due to the small sample size non-normal distribution of the data was assumed and the groups were compared using the Wilcoxon signed rank test. All results are reported as median with range. We also analysed the correlation between the number of failure cycles and the BMD values using linear regression analysis.

## Results

### Varus bending testing

In the varus bending testing all specimen pairs failed by loosening of the screws in the humeral head and subsequent in varus tilting of the head relative to the plate. The group of the LS 3.5 screws reached 2901 (range 601 to 5201) load cycles until failure of the fixation occurred, while the group of the DLS 3.7 screws failed after 3731 (range 2001 to 5601) cycles. This corresponds to a median loading of 195 N and 235 N for the LS 3.5 and the DLS 3.7 group, respectively.

**Fig. 5:**
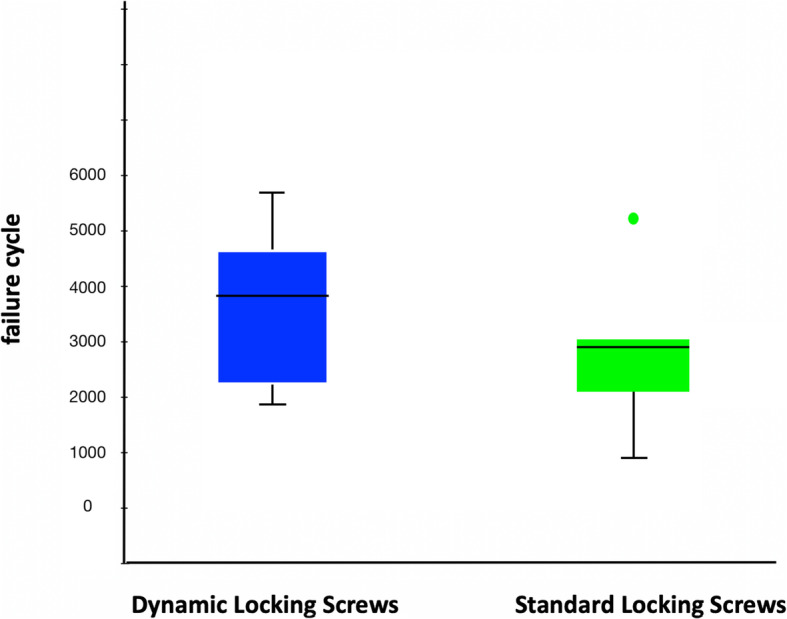
Failure cycle of the bending test in boxplot for DLS and standard (LS) screws

The comparison of both test groups shows that each test specimens with DLS 3.7 screws sustained a higher number of load cycles until failure than the corresponding contralateral humeri with the standard (LS 3.5) screws (Fig. 5). Failure cycle using DLS 3.7 screws were 400–1600 cycles higher than the failure cycle using LS 3.5 screws. The difference between the two groups were significant (*p* = 0.028).

### BMD

The specimens showed a strong positive correlation (*R*^*2*^ = 0.950, *p* = 0.005) between the BMD and the failure cycle in varus bending in the DLS-group. We found strong positive correlation (*R*^*2*^ = 0.950, *p* = 0.005) in the group of the DLS. This correlation between BMD and failure cycle is weaker in the LS-group. (*R*^*2*^ = 0.427, *p* = 0.232). There was no significant difference of BMD between groups.

### Axial rotation testing

**Fig. 6:**
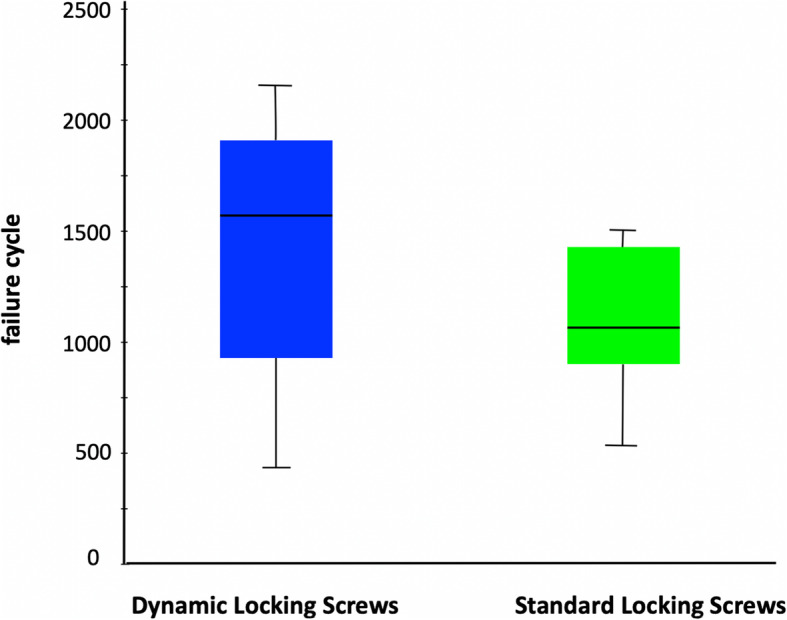
Failure cycle of the torsion test in boxplot for dynamic (DLS) and standard (LS) screws

In the torsional load case (axial rotation) all specimens failed by loosening of the screws in the humeral head and subsequent excessive torsion of the head relative to the plate. The LS-group reached a median of 1101 (range 501 to 1501) load cycles until failure of the fixation occurred, while the DLS-group failed after 1401 (range 401 to 2201) load cycles. These failure cycles correspond to a median torque of 3.25 Nm and 4.09 Nm for the LS- and the DLS-group, respectively (Fig. 6).

The comparison of both test groups showed that of the six specimen pairs, the DLS-group showed 3 times a higher number of load cycles and the LS-group showed twice a higher number of load cycles until failure, while one specimen pair showed identical failure cycles. There was no statistical difference between the two gest groups (*p* = 0.225). So all test specimens with the dynamic fixation sustained a higher or similar number of load cycles until failure than the corresponding contralateral humeri with conventional standard locking screws.

The specimens in both groups showed only a weak correlation between the BMD and failure cycle (LS-group: *R*^*2*^ = 0.483, *p* = 0.126; DLS-group: *R*^*2*^ = 0.196, *p* = 0.379).

### FEM analysis of screw load distribution in PHILOS using standard or dynamic locking screws

**Fig. 7:**
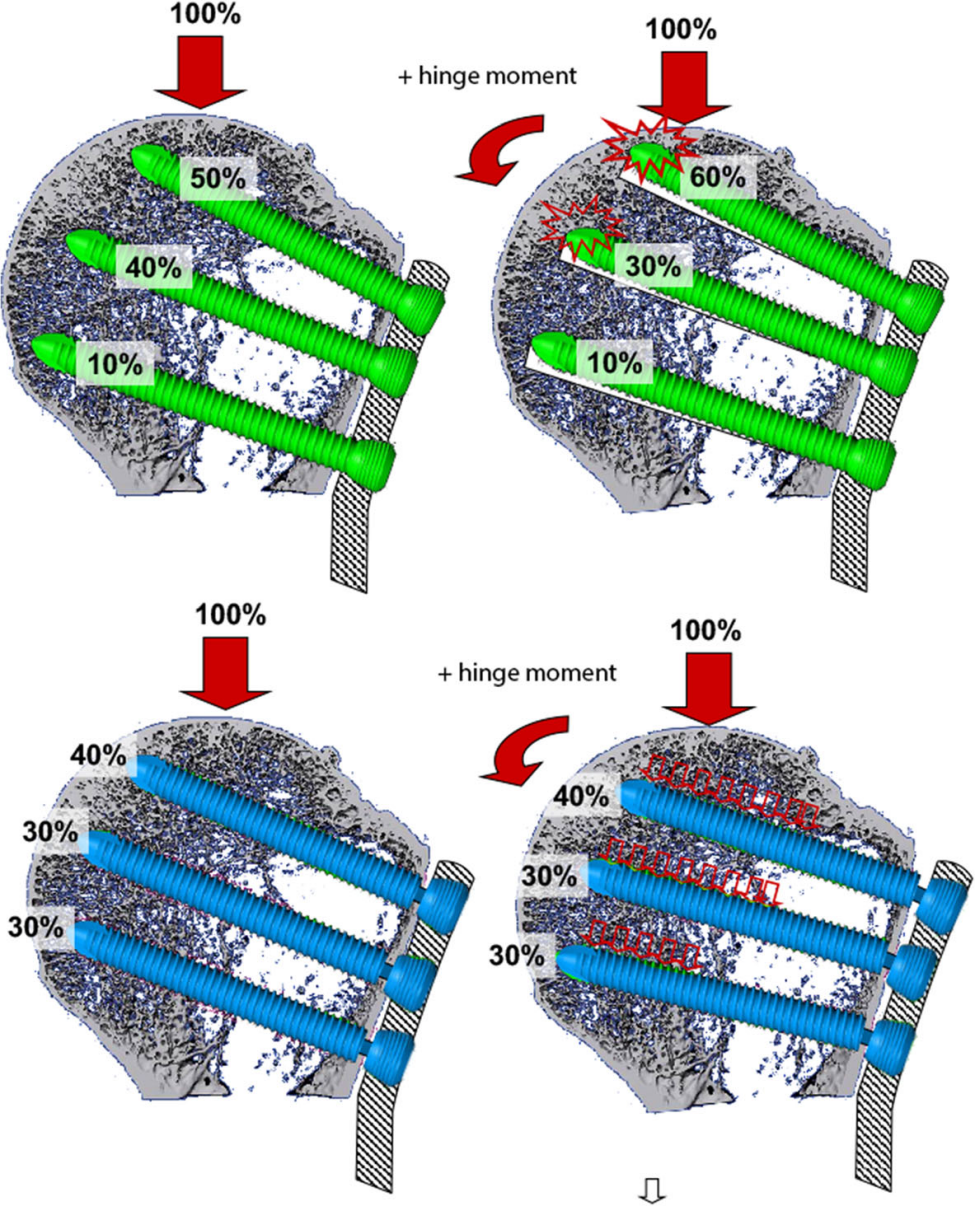
Screw load distribution with standard locking screws (green) versus dynamic locking screws (blue)

We found a lower load maximum and a more evenly distributed loading pattern in the model for dynamic locking screws compared to standard locking screws (Fig. 7).

## Discussion

The most important finding of this biomechanical study demonstrated higher loads to failure for dynamic locking screws (DLS) in comparison with standard locking screws (LS). Common treatment options for proximal humeral fractures are still associated with high complication rates [2, 18, 19]. In search for improving the biomechanical properties of fracture fixation in healthy and osteoporotic bone, DLS were developed [29]. The general mechanical properties of DLS had been studied earlier in biomechanical studies [9, 10]. In angle stable plate osteosynthesis of the proximal humerus, it was clinically demonstrated that the mechanical load is distributed more equally among the screws, if DLS are used instead of standard LS (Fig. 7) [12].

With the present biomechanical study, we verified a higher failure load using DLS versus LS in angle stable plate osteosynthesis of the proximal humerus in a fracture model. This might lead to reduced secondary dislocations and improved or at least more consistent clinical outcomes after osteosyntheses of proximal humeral fractures.

Dynamic PHILOS plate fixation leads to better screw anchorage in proximal humeral fractures in comparison to the conventional fixation. By using the dynamic fixation technology 400–1600 cycles more could be applied until failure occurred, whereas the conventional locking plate fixation failed significantly earlier in varus bending testing. These findings were observed both in proximal humeri with normal BMD and in osteoporotic bone with reduced BMD.

These positive findings can be explained by the special design of the dynamic screws, as they enable a relative motion of 0.2 mm between the head of the screw and the sleeve of the screw [9, 10]. Thereby, a more homogenous intra- and an inter-screw load distribution of the pin-sleeve-construct of the DLS 3.7 can be achieved. This has two advantages: 1) Intra-screw: the force is distributed over a longer distance along the screw axis. 2) Inter-screw: because of the reduced rigidity of the plate-screw construct, a certain damping effect occurs.

The motion between the screw and the plate leads to a more homogeneous load distribution over all implanted screws. By using the standard locking screws (LS 3.5), 60% of the peak stress applies to the two proximal plate-screws (A position of the PHILOS plate). With the dynamic locking screws (DLS 3.7), the stress can be reduced to 40% in these particular screws [12].

The design of the screw tip of the DLS 3.7 is likely to also have a positive effect on screw anchorage and might help to prevent the screw from cut-out. Compared to LS 3.5 (3.5 mm) the DLS 3.7 (3.7 mm) has a round tip and fewer sharp edges at the end of the screw. This probably contributes to a reduction of peak interface stresses between the tip of the screw and the bone, preventing screw loosening and failure.

DLS probably don’t achieve the same rigid biomechanical properties as cement augmented screws [23, 28]. But the idea of a more dynamic osteosynthesis demonstrated its beneficial biomechanical principles in the setup of this study and might contribute to improve the failure rates in standard fracture treatments, especially in scenarios with poor bone substance like proximal humeral fractures.

Plate fixation using dynamic locking screws for the treatment of proximal humerus fractures demonstrated more load cycles until failure compared to standard locking plate osteosynthesis. In the clinical setting, this could provide a safer osteosynthesis and less secondary failures. So, patients could potentially benefit from the clinical application of dynamic locking screws in proximal humerus plate osteosynthesis. Although we demonstrated successful fracture fixation, further studies are needed before DLS can be recommended as standard implants in proximal humeral plate osteosynthesis.

## Conclusions

In this biomechanical study using a model of proximal humeral plate osteosynthesis, we demonstrated higher loads to failure for dynamic locking screws (DLS) in comparison with standard locking screws (LS). This might help to reduce secondary dislocations. A proximal humerus plate with DLS could potentially present a biomechanically optimized implant for clinical application in patients with fractures of the proximal humerus, especially in comminuted and osteoporotic situations.

## Data Availability

The datasets generated and analysed during the current study are available from the authors on reasonable request.
